# Report of 13-year survival of patients with colon and rectal cancers; lessons from Shiraz colorectal cancer surgery registry system of a level three medical center

**DOI:** 10.1186/s12893-022-01591-2

**Published:** 2022-04-15

**Authors:** Ali Reza Safarpour, Alimohammad Bananzadeh, Ahmad Izadpanah, Leila Ghahramani, Seyed Mohammad Kazem Tadayon, Faranak Bahrami, Seyed Vahid Hoseini

**Affiliations:** Colorectal Research Center, Shiraz University of Medical Sciences, Shiraz, Iran

**Keywords:** Colorectal neoplasms, Colonic Neoplasms, Rectal neoplasms, Survival analysis, Kaplan-Meier Estimate

## Abstract

**Background:**

Colorectal cancer (CRC) is the second most common cancer in women and the third most common cancer in men worldwide, with an increasing trend in its incidence in Asian countries. In the present study, we aimed to describe the 13-year results of patients with CRC based on the Shiraz Colorectal Cancer Surgery (SCORCS) registry system in patients with a pathologically confirmed diagnosis of colon cancer (CC) and rectal cancer (RC) undergoing surgery.

**Methods:**

Between 2007 and 2020, 811 patients, including 280 patients with CC and 531 patients with RC, registered in SCORCS, were included in the present study. The information collected for this study included demographic characteristics of the patients, primary clinical presentations, laboratory findings before surgery, radiologic and colonoscopy results, and surgical procedures. Death was confirmed by the physician as “CRC-related”. The data were analyzed by SPSS software version 21; life table and Kaplan-Meier curve were used for evaluating the overall survival, recurrence, and metastasis rates and Log-Rank test or Breslow test to check significant differences between the subgroups. The Cox proportional regression model was fitted to evaluate the prognostic factors of survival recurrence and metastasis.

**Results:**

Laparoscopy was performed in 60% of patients (66% in RC and 51% in CC), laparotomy in 32% (27% in RC and 41% in CC), and 7% required conversion. The median time of follow-up was 29 months in all patients; 28 months in patients with RC, and 33 months in patients with CC; 1, 3, and 5 years’ survival rate was 90, 70, and 63% for all the patients, 89%, 67%, and 58% for RC and 90%, 74%, and 71% for CC, respectively (P = 0.009). The Cox regression analysis revealed tumor stages II, (P = 0.003, HR:2.45, 95% CI;1.34–4.49), III, (P ≤ 0.001, HR:3.46, 95% CI;1.88–6.36) and IV, (P ≤ 0.001, HR:6.28, 95% CI;2.73–14.42) in RC and stage IV, (P = 0.03, HR:9.33, 95% CI;1.1-76.37) in CC were the significant survival prognostic factors. The metastasis and recurrence of the tumors occurred earlier in patients with RC than CC (P = 0.001 and 0.03, respectively).

**Conclusions:**

Long-term follow-up of patients with CRC in an Iranian population indicated the significance of screening for diagnosis of early stages and improved survival of the patients.

## Background

Colorectal cancer (CRC) is currently the second most common cancer in women and the third most common cancer in men in the world [1]. According to the World Health Organization, 1.80 million new cases of CRC were diagnosed in 2018 [2, 3]. In recent years, the prevalence of CRC has increased in Asian countries, turning it into one of the most important leading causes of death on the continent [4]. In Iran, CRC has been identified as the fourth most common cancer in men and the third most common cancer in women [5], with an increasing trend in its incidence [6].

Although colon and rectum are supposed as parts of a single organ, the risk of developing rectal cancer (RC) per cm is generally four times higher than colon cancer (CC); accordingly, because of the differences in carcinogenesis, clinical presentations, growth pattern, metastatic patterns [7], treatment outcome, and survival rates [8] between CC and RC, it has been suggested to abandon the term CRC [9]. CC and RC also differ in terms of the effect of preventive measures, including exercise, body mass index (BMI), reduced energy intake, and medications, such as COX-2 inhibitors or aspirin, on the risk of cancer incidence [9, 10].

Currently, the only effective treatment for both CC and RC is surgical excision of the tumor and local invasions, including regional neuronal and lymphatic drainage areas, while the surgical procedure is more challenging in RC, especially tumors close to the sphincter muscles, resulting in a higher rate of recurrence and morbidity/mortality [11]. On the other hand, the surgical procedure is also challenging in patients with stage IV CC, because of the need for multi-organ resection [12]. Laparoscopic resection is more frequently practiced, especially in patients with CC; however, the choice of surgical method depends on the patients’ conditions and physicians’ preference [13, 14]. Multimodal treatment, including adjuvant chemotherapy or chemo-radiotherapy, is also used for both CC and RC [15], while not all patients require adjuvant therapy, and it is suggested to be used for high-risk groups [16]. Pathology also has a significant role in diagnosis, disease staging, surgical margins, lymphovascular and perineural invasion, and evaluating the treatment response after surgery [17].

Valid results about the outcomes of patients with cancer are based on data collected by registry systems, and several countries have established cancer registry systems for this purpose [18]. Several cancer registry systems have also been launched in Iran, which record the data collected from one or a few provinces in the country, covering less than 20% of the whole population with limited data recorded [19, 20]. A review of studies also shows that a bunch of cancer reports is based on hospital-based cancer registry (HBCR) systems because of the lack of nationwide registry systems in Iran and other countries [21]. In this study, we analyzed the data collected by the Shiraz colorectal cancer surgery (SCORCS) registry system, which records complete information of all patients with CRC in a tertiary referral hospital, affiliated to Shiraz University of Medical Sciences, Shiraz, Iran, since Sep. 2007 by an expert registry team under the supervision of specialists. Therefore, in the present study, we aimed to report the five-year survival, recurrence, and metastasis rates and their predictors during the 13 years of data registry in addition to describing their demographics of patients with pathologically-confirmed diagnosis of CC and RC undergoing surgery.

## Methods

The “SCORCS” registry is a web-based electronic registry, established in Jan. 2007 at “Shahid Faghihi” Hospital, affiliated to Shiraz University of Medical Sciences, Shiraz, Iran, as a level three highly specialized center for surgical treatment, to collect data of patients with CC or RC. Diagnosis of CRC was confirmed based on the pathological report of the examination of the sample taken from the patients’ intestinal tissue before the surgery. RC was defined as tumors between the anorectal ring and sigmoid take-off (i.e. the junction between the sigmoid mesocolon and mesorectum), detected on cross-sectional imaging. Additional histological information was prepared after tumor resection by an expert pathologist and then recorded in the database by the registry personnel. We comprised all recurrences as presence of tumor at anastomotic or regional area during follow up. Metastasis was defined as involvement of liver, lung or any other distant organs.

A total of 811 patients, including 280 patients with CC and 531 patients with RC have been registered and included in the present study.

Each patient admitted to the colorectal department of the Shahid Faghihi Hospital with the confirmed diagnosis was introduced by the head nurse of the ward to the data registrar team, who registered the patients’ information into the SCORCS of the hospital, under the supervision of one epidemiologist (ARS) and one colorectal surgeon (AB) for data clearance and continuous validation of data. At admission, the patients signed the informed consent form and provided their contact information (including phone and cell phone number, fax, email and WhatsApp, address and cell phones of first relations), in order to complete the database. The registry team also encouraged the patients to regularly visit to the clinic and help to complete the database.

The information collected from the registry for this study included demographic characteristics of the patients (including sex, age, ethnicity, marital status, and educational level), blood group, smoking, use of hookah, and substance abuse, use of oral contraceptives (OCP) in women, date of cancer diagnosis (according to pathology report), primary clinical presentations (including change in bowel habit, diarrhea, constipation, rectal bleeding, and anorexia), and laboratory findings before surgery (including serum levels of hemoglobin, protein, albumin, alkaline phosphatase, and carcinoembryonic antigen. The results of radiological (computed tomography [CT] scan) and colonoscopy examinations as well as the type of surgery performed, were also recorded. The staging was performed based on the American Joint Committee on Cancer Staging (stages I–IV; 7th edition) [22].

Follow-ups of the patients were performed 1, 2, and 4 week(s) after the surgery, after 3 months, 6 months, 1 year, and then every year till 10 years or occurrence of death, confirmed by the physician as “CRC-related”. Before survival analysis, in this study we excluded two patients who were died due to causes other than their carcinoma, one with rectal carcinoma (patient number: 469 in dataset, death cause: car accident) and also one patient with colon carcinoma (patient number: 751 in dataset, death cause: myocardial infarction). For this reason, “overall survival” which was reported in this study was much closed to “CRC-related survival”. All patients underwent elective surgery with bowel preparation the day before the operation. The preferred technique for the operation was laparoscopy.

This study has been registered with the registration number 91-01-69-4803 in the office of the Institute of Board Review of Shiraz University of Medical Sciences and approved by the Ethics Committee of Research under the number: IR.SUMS.REC0.1392.4803.

### Statistical analysis

The variables were described using number (percentage) and mean ± standard deviation (SD). The Pearson’s Chi-square test or Fischer’s exact test were used for comparison of categorical variables, and two-sided independent samples *t* test was used for comparison of quantitative variables between the groups. The normal distribution of quantitative data was checked by the Shapiro Wilks test for normality. The P values greater than 0.05 were considered normally distributed variables. The life table and Kaplan-Meier curve were used to evaluate the overall survival rate during the follow-up period. The Log-Rank test or Breslow test was performed to check significant differences between the subgroups. The Cox proportional regression analysis was performed to find the predictive factors of survival, recurrence, and metastasis. The significance level was set at the point of < 0.05. IBM SPSS Statistics for Windows (Released 2012. Version 21.0. Armonk, NY: IBM Corp) and Stata software version 13 were used for analysis.

## Results

A total 811 patients with CRC, including 531patients with RC and 280 patients with CC, were included in this study. The basic demographic characteristics of registered patients with CC or RC are demonstrated in Table 1. As shown in this table, 42% of patients with RC and 47% of patients with CC were women. 40% of the patients with RC and 43% of the patients with CC were overweight and obese. There was no significant difference between the patients with CC and RC in terms of the sex distribution, mean age, educational level, and marital status (P > 0.05; Table 1. Most of the patients were married, and most of the ethnicities included Fars and Lor, as shown in Table 1.

**Table 1:** Demographic characteristics of patients with colon or rectum carcinoma

Variable	Categories	Rectal cancer (n=531)	Colon cancer (n=280)	Total (n=811)	P value
Age, Mean±SD		56.93±13.43	57.08±14.61	57.03±13.85	0.88^*^
Sex, N (%)	Female Male	222 (42%) 309 (58%)	132 (47%) 148 (53%)	355 (44%) 460 (56%)	0.14^†^
Body mass index (kg/m^2^), N (%)	Underweight Normal weight Overweight Obesity	46 (9%) 269 (51%) 156 (29%) 57 (11%)	32 (11%) 123 (44%) 88 (31%) 34 (12%)	78 (10%) 395 (48%) 245 (30%) 91 (11%)	0.28^†^
Educational level, N (%)	Illiterate Primary school High School Post graduate Academic degree	150 (28%) 4 (1%) 126 (24%) 191 (36%) 60 (11%)	64 (23%) 2 (1%) 82 (29%) 100 (36%) 32 (11%)	216 (27%) 6 (1%) 208 (26%) 292 (36%) 93 (11%)	0.37^†^
Marital status, N (%)	Divorced Married Single Widowed	2 (0%) 502 (95%) 24 (5%) 3 (1%)	0 (0%) 266 (95%) 11 (4%) 3 (1%)	2 (0%) 722 (95%) 35 (4%) 6 (1%)	0.85^†^
Ethnicity, N (%)	Arab Balooch Fars Kurd Lor Other Turk	16 (3%) 0 (0%) 441 (83%) 3 (1%) 48 (9%) 4 (1%) 19 (4%)	6 (1%) 2 (1%) 234 (84%) 2 (1%) 24 (9%) 2 (1%) 10 (3%)	22 (0%) 2 (3%) 679 (83%) 5 (1%) 72 (9%) 6 (1%) 29 (4%)	0.74^†^
OCP consumption, N (%)	No Yes	136 (61%) 85 (38%)	86 (65%) 43 (%34)	222 (63%) 131 (37%)	0.33^†^
Smoking, N (%)	Current smoker Ex-Smoker Non-smoker	71 (13%) 105 (20%) 355 (67%)	34 (12%) 51 (18%) 195 (70%)	105 (13%) 156 (19%) 554 (68%)	0.72^†^
Type of smoking, N (%)	Cigarette	101 (61%)	58 (37%)	159 (100%)	0.89^†^
	Opium	40 (67%)	20 (33%)	60 (100%)	0.65^†^
	Hookah	74 (76.3%)	23 (23.7%)	97 (100%)	0.007^†^

*The results of independent samples *t* test, ^†^The result of Chi-square test

**Fig. 1:**
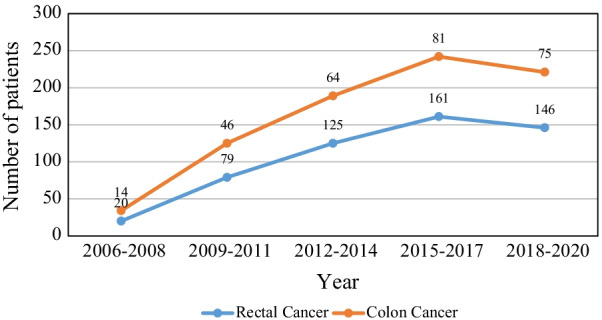
Trend of number of surgery in patients with colon and rectal carcinoma, 2006–2020

**Fig. 2:**
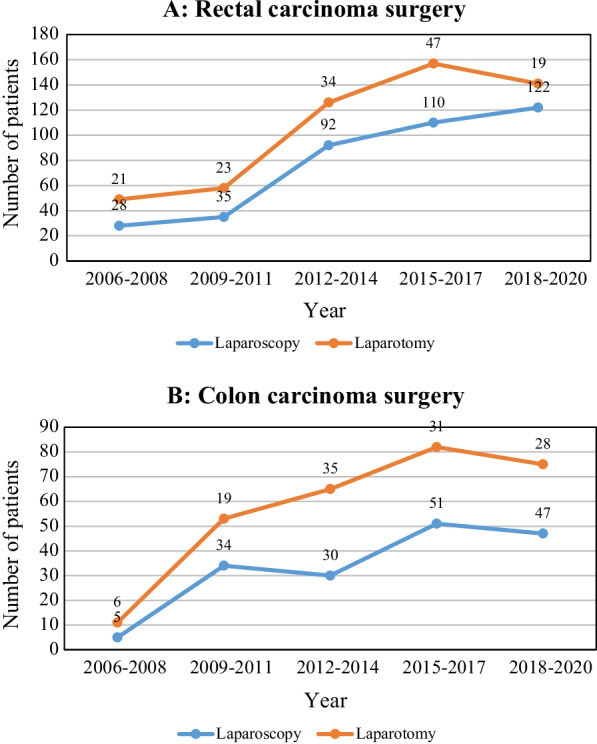
Trend of laparoscopic versus laparotomy surgery in patients with rectal **A** and colon **B** cancer, 2006–2020

Studying the frequency of clinical presentations at diagnosis in patients with CC and RC (Table 2) demonstrated rectal bleeding as the most common clinical presentation in patients with RC (79%) and change in bowel habit in patients with CC (60%). The results of laboratory, radiological, and colonoscopy examinations are also shown in Table 2. Based on these results, the amount of hemoglobin, serum protein, and serum albumin of the two groups had a statistically significant difference (P < 0.001, P = 0.02, and P = 0.04, respectively). The most common blood group in both cancer groups was O, and the least common blood group was AB (Table 2). Luminal obstruction because of the tumor has also been reported as the most common colonoscopy finding in patients in both cancer groups. The number of total surgical procedures performed during the study period is shown in Fig. 1. Among all patients, 492 patients underwent laparoscopy (60%; 349 [66%] in RC and 143 [51%] in CC), 257 patients underwent laparotomy (32%; 143 [27%] in RC and 114 [41%] in CC), and 61 patients required conversion of laparoscopy to laparotomy (38 [7%] in RC and 23 [8%] in CC). The trend of changes in the frequency of laparoscopy and laparotomy along the follow-up period is shown in Fig. 2. Most patients with RC underwent low anterior resection (N = 371, 75.4%), and the rest underwent abdominal perineal resection. The frequency of the type of surgery in patients with CC was as follows: 52 (18.5%) sigmoidectomy, 112 (40%) right hemicolectomy, 76 (27%) left hemicolectomy and 40 (14.5%) total colectomy.

**Table 2:** The frequency of clinical presentation and the results of laboratory and radiological examinations of patients with colon or rectum carcinoma before surgery

Variable	Rectal cancer (n=531)	Colon cancer (n=280)	Total (n=811)	P value
GI symptoms				
Change in bowel habit	137 (26%)	167 (60%)	304 (38%)	<0.001^†^
Constipation	212 (40%)	119 (42%)	331 (41%)	0.44^†^
Diarrhea	97 (18%)	27 (10%)	124 (15%)	0.001^†^
Bloody stool	68 (70%)	16 (59%)	84 (68%)	0.30^†^
Non-bloody stool	29 (30%)	11 (41%)	40 (32%)	0.27^†^
Anorexia	46 (9%)	42 (15%)	88 (11%)	0.005^†^
Rectal bleeding	418 (79%)	118 (42%)	536 (66%)	<0.001^†^
Lab. findings				
Hemoglobin (g/dL), Mean±SD	12.36±1.86	11.36±2.41	12.03±2.12	<0.001^*^
Alkaline Phosphatase (IU/L), Mean±SD	221.98±98.34	225.14±116.61	222.41±104.79	0.68^*^
Serum protein (g/dL), Mean±SD	4.2±3.5	3.6±3.5	4±3.5	0.02^*^
Serum albumin (g/dL), Mean±SD	2.5±2	2.2±2	2.4±2	0.04^*^
Carcinoembrionic antigen (ug/L), Mean±SD	1.68±7.52	2.7±13.65	2.06±10.07	0.20*
Blood Groups				
A+	90 (18.9%)	30 (6.3%)	120 (25%)	
A−	8 (1.7%)	2 (0.4%)	10 (2.1%)	
AB+	12 (2.5%)	10 (2.1%)	22 (4.6%)	
AB−	1 (0.2%)	0 (0%)	1 (0.2%)	0.19^†^
B+	69 (14.5%)	33 (6.9%)	102 (21.4%)	
B−	5 (1%)	1 (0.2%)	6 (1.2%)	
O+	130 (27.3%)	71 (15%)	201 (42.3%)	
O−	7 (1.5%)	6 (1.3%)	13 (2.8%)	
CT Scan				
Normal CT scan results	45(8%)	13(5%)	58(13%)	0.04^†^
Abnormal CT scan results	250(47%)	134(48%)	384(87%)	
Findings during colonoscopy				
Hemorrhoid during colonoscopy	36 (7%)	19 (7%)	55 (7%)	
Polyp during colonoscopy	54 (10%)	32 (12%)	86 (10%)	0.07^†^
Luminal obstruction due to the tumor	57 (11%)	44 (16%)	101 (12%)	
Staging				
No residual	31 (6.1%)	7 (2.7%)	38 (4.9%)	
Stage I	129 (25.3%)	47 (17.8%)	176 (22.8%)	
Stage II	142 (27.9%)	87 (33.0%)	229 (29.6%)	<0.001^†^
Stage III	118 (23.2%)	91 (34.5%)	209 (27.0%)	
Stage IV	18 (3.5%)	18 (6.8%)	36 (4.7%)	

*The results of independent samples *t* test, ^†^The result of Chi-square test

**Fig. 3:**
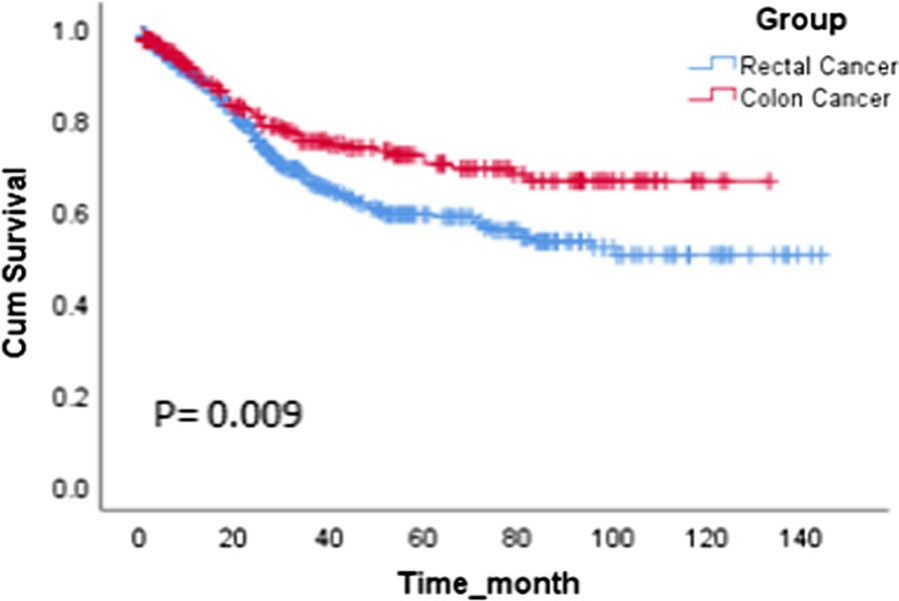
Kaplan-Meier curves of estimated overall survival rate of patients with colon or rectal cancer during 13 years of follow-up after surgery

**Fig. 4:**
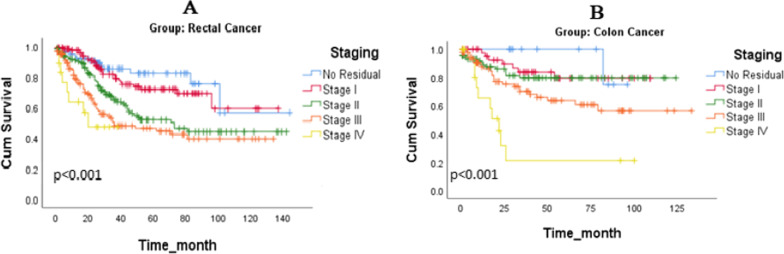
Comparison of survival rate of patients with rectal cancer **A** and colon cancer **B** in different stages during 13 years of follow-up after surgery

The median time of follow-up for all patients was 29 months (IQR: 13–59), for patients with RC was 28 months (IQR; 14.50-55.05), and for patients with CC was 33 months (IQR; 9.25–65.50). The life-table analysis revealed that 1-, 3-, and 5-year survival of all patients were 90%, 70%, and 63%; for the patients with RC were 89%, 67%, and 58%, and for the patients with CC were 90%, 74%, and 71%, respectively, with a statistically significant difference between the groups, based on Kaplan-Meier method and Log-Rank test results (P = 0.009; Fig. 3). The survival rate of patients at different disease stages after surgery was as follows: 90%, 71%, 51%, and 46% in patients with RC at no residual, stages I, II, and III, respectively, and 100%, 79%, 78%, 63%, and 23% in patients with CC at no residual, stages I, II, III, and IV, respectively. As shown in Fig. 4, the survival rate of different stages of RC (Fig. 4 A) and CC (Fig. 4B) decreases with increasing tumor stages. The Cox regression model with backward LR, was run for RC and CC separately. In both RC and CC, the only variable wich remained in the final model was stage of tumor as a significant predictor of survival. In RC, tumor stages II, (P = 0.003, HR = 2.45, 95% CI: 1.34–4.49), III, (P < 0.001, HR = 3.46, 95% CI: 1.88–6.36), and IV (P < 0.001, HR = 6.28, 95% CI: 2.73–14.42) and in CC, tumor stage IV (P = 0.037, HR = 9.34, 95% CI: 1.14–76.37) were identified as a significant predictor of patients’ survival. The effect of confounding variables were adjusted in two models which were not significant. These Variables in model for RC were, “changes in bowel habit”(p = 0.31 ), “diarrhea” (p = 0.42 ), “anorexia(p = 0.37 )”, “rectal bleeding” (p = 0.60 ), “hemoglobin” (p = 0.97 ), and “CT scan findings” (p = 0.18 ) and in CC model were, “changes in bowel habit”(p = 0.31 ), “diarrhea” (p = 0.26 ), “anorexia” (p = 0.57 ), “rectal bleeding” (p = 0.68 ), “hemoglobin” (p = 0.29 ), and “CT scan findings” (p = 0.79 ).

**Fig. 5:**
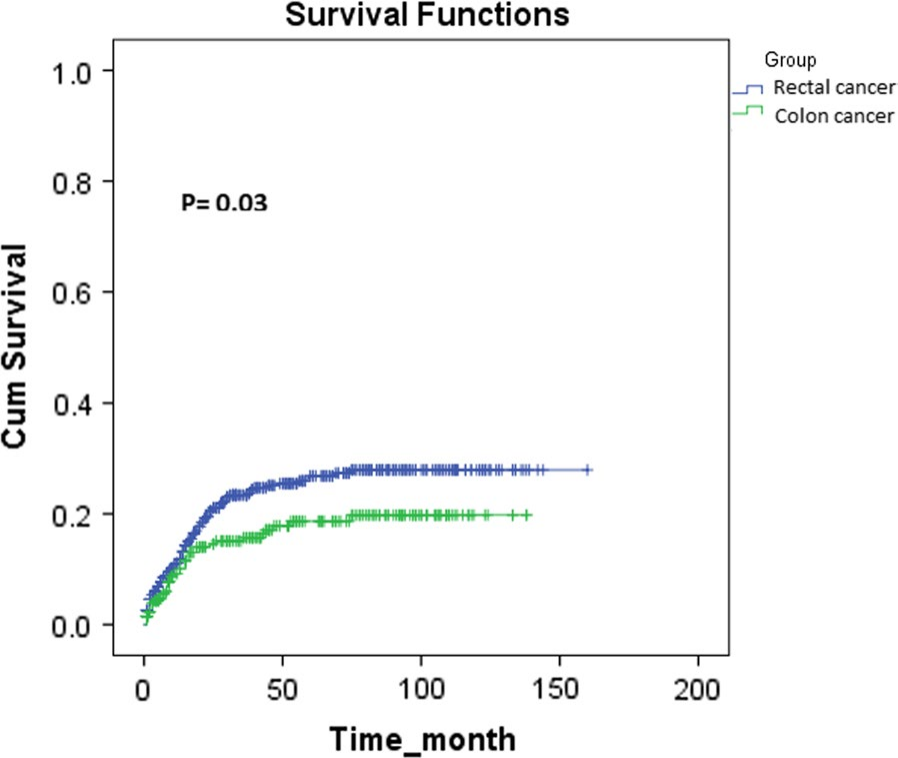
Comparison of time to metastasis of patients with rectal cancer and colon cancer

**Fig. 6:**
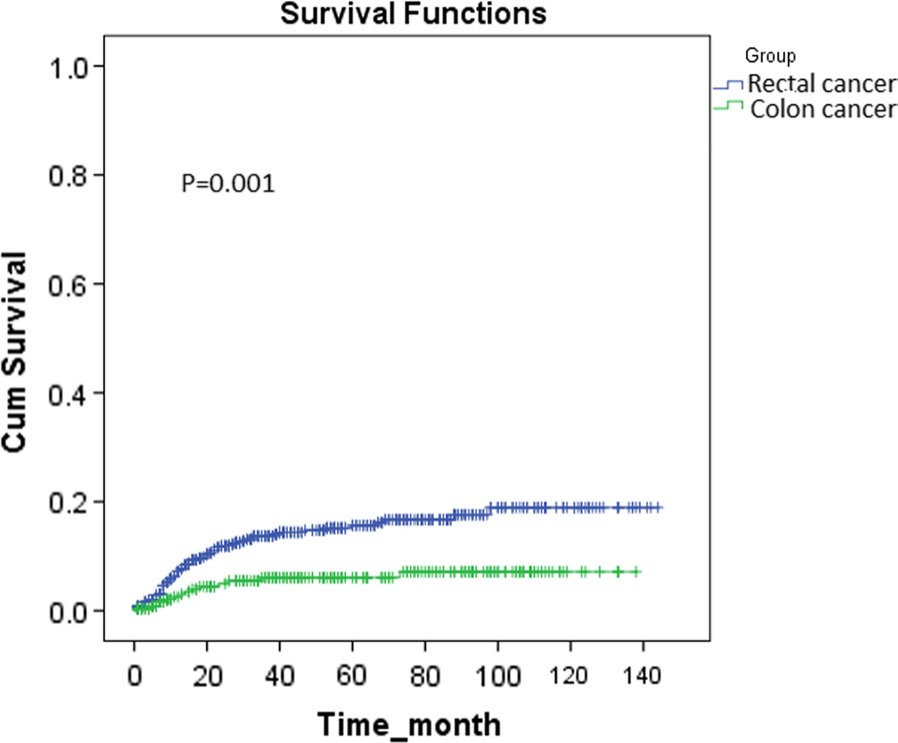
Comparison of time to recurrence of patients with rectal cancer and colon cancer

1-, 3-, and 5-year proportion of metastasis-free in RC were 94%, 87%, and 63%, and in CC were 94%, 79%, and 77%, respectively, with a significant difference between RC and CC (P = 0.03, Fig. 5). Also, 1-, 3-, and 5-year proportion of recurrence-free in RC were 98%, 86%, and 81%, and in CC were 100%, 95%, and 92%, respectively, with a significant difference between RC and CC (P = 0.001, Fig. 6). The results of Kaplan-Meier analysis and Log-Rank test revealed that metastasis and recurrence of the tumors occurred earlier in patients with RC than CC (P = 0.001 and 0.03, respectively).

## Discussion

In the present study, we described the results of analyzing the 13-year registration of patients with CRC (34.52% CC and 65.47% RC) in a tertiary referral hospital, Shiraz, Iran. The results of another Iranian study on the national cancer registry system, about 62% of CRC patients had CC [23], which is contrary to the results of the present study. Others have also reported the incidence rate of CC two times higher than RC [24, 25], which contradicts the patient proportion in the present study. The higher frequency of patients with RC in our study could be related to the fact that our hospital is a referral center. The differences in the risk factors in the target population, particularly in dietary habits of different ethnic groups, may also influence this proportion [26].

The main outcome of the present study was patient’s survival rate during the 5-year follow-up, and the results showed different survival rates according to the type of CRC and disease stage. As shown, the 5-year survival rate was 63% for patients with CRC, 58% for patients with RC, and 71% for patients with CC, and patients with RC had an earlier metastasis and recurrence, as well as lower metastasis-free and recurrence-free rates, compared with the patients with CC. In a previous study on the same database, SCORCS, the results of following 346 patients with CRC (until 2017) showed 1, 3, and 5 year survival rates at 93, 71, and 65% [27], which is similar to the results of the current study. In another study in Shiraz, the overall 5-year survival rate of the patients with CRC was 58.5% [28], which seems lower than that reported in the developed countries like Germany [29]. An analysis of 837 Chinese patients with CRC has also shown a 5-year survival of 69% for CC and 66% for RC [30], which is different from that in the present study. This difference could be because of the different frequencies of the disease stage in the study populations, as patients with national screening systems tend to have lower rates of tumor stage IV and higher rates of stage I [31]. The significant effect of the tumor stage on patients’ survival, presented in the current study, is in line with the results of previous reports [32, 33]. Furthermore, the variety of the classification system used and the stages included in the studies are considered other factors for the different survival rates reported [34]. Another influential factor for the difference in the survival rates reported is the number of follow-up visits [35]. A difference in other factors affecting the survival rate, including race, access to high-quality health care, dietary intake, and screening, can also result in different rates among studies [36]. In addition to the factors mentioned-above, another important factor for the lower survival rate of RC in our country could be related to the shame the patients have from the anal area of their body that may result in late referral of the patients to the physician and higher disease development [37].

The trend of patients’ survival rates during the study period in the present study shows a steady decreasing trend for both CC and RC. In the study by van der Sijp on 767 patients with CC and 272 patients with RC who underwent resection, a similar trend is observed for both groups [25], which is consistent with the results of the present study. A notable finding in the present study was the number of surgical procedures, and the results showed an increasing trend until 2015–2017, which became steady thereafter, which can be attributed to the recent pandemic of coronavirus disease-2019 (COVID-19) around the world as well as in our country, which led to decrease patients who referred to the hospital for treatment of colorectal tumors. This issue has been pointed out as a considerable issue in the treatment of patients with CRC [38] and other cancers [39], which emphasizes the necessity of paying greater attention to this issue and planning new strategies for fixing this problem and its adverse effect on patients’ survival.

Studying the demographic characteristics of the patients showed that more than half were men in both groups. Other studies have also shown a higher incidence of CRC in the male sex, suggesting it as a strong predictor of CRC [40, 41], attributed to the role of male sex hormones in CRC [42, 43]; however, Cox regression analysis did not identify the sex as a significant predictor of survival in the present study, which showed that the difference in the frequency of male and female did not affect the survival rates in our results. The mean age of diagnosis reported in the present study was about 57 years in both groups, which may vary from other studies, according to the disease stage at the time of diagnosis and the presence of risk factors of early- or late-onset CRC in that specific population [44]. The risk factors evaluated in the present study included obesity, smoking, OCP use, and ethnicity. Cigarette smoking (current and former) has been suggested as a strong predictor of CRC incidence and mortality [45, 46], which was very low in our study (12–13% were current smokers and 18–20% ex-smoker). Hookah use, on the other hand, was different between patients with CC and RC in the present study, and most patients who used hookah had RC; however, a meta-analysis of studies failed to show hookah use as a significant risk of CRC [47]. Furthermore, obesity and overweight were observed in about 40% of patients of the present study, which is consistent with the suggestion of a higher incidence of CRC by the increase in BMI [48]. One of the modifiable risk factors that has been considered less frequently is OCP use that was reported in about one-third of the female population in our study. As the results of a meta-analysis of 15,790 patients with CRC have shown, OCP use has a predictive role against the risk of CRC incidence and higher tumor stages and is thus suggested to be used in women with a high risk of CRC [49], while further studies are required in this regard. Although some studies have reported different risk factors between RC and CC [8, 50], we did not find a difference in most of the studied risk factors between the patients with RC and CC, and none had a significant effect on patients’ survival. The type of surgery also had no effect on patients’ survival, which is in line with a previous report [51]. Among the wide range of variables studied, only the tumor stages II, III, and IV in patients with RC and stage IV in patients with CC, were identified as the predictor of survival.

One of the main strengths of the present study was the validity of the recorded data in the SCORCS registry system, as the data has been recorded by an expert team, reviewed for data clearance, and was continually under the supervision of patients’ physicians and specialists. Also, we reported the results of pathologically-confirmed cases based on total patients with CRC, as well as for the patients with colon and RC separately, in order to be comparable with studies on either CRC or CC/RC. However, the study also had some limitations. One of the limitations was related to the access to the patients after surgery that resulted from the loss to follow-up cases. Another limitation is related to the generalizability of the results, as the data were collected from one referral medical center that may be applicable to patients in this city and the surrounding cities.

## Conclusions

The long-term follow-up of patients using a valid registry system in the present study provided valuable information about the demographic and clinical characteristics of patients with CRC as well as RC and CC, separately, as well as the frequency of risk factors in these patients. We also calculated 1-, 3-, and 5-year survival rate for both groups, while comparing the survival rates in the present study with other studies showed a lower survival rate for RC in our study, which has to be further investigated in future studies. Surgery was performed for all patients in the present study, and the trend in the number of surgical procedures showed a cease in the increasing trend in recent years, which requires the attention of policymakers to encourage patients to refer to the hospital during COVID-19 pandemic. Although the present study provided valuable information, it is suggested that future studies report the detailed information of patients with cancer, based on national registries.

## Data Availability

The datasets used and/or analysed during the current study are available from the corresponding author on reasonable request.

## Abbreviations

BMI: Body mass index
CC: Colon cancer
CRC: Colorectal cancer
CT: Computed tomography
HBCR: Hospital-based cancer registry
OCP: Oral contraceptives.
RC: Rectal cancer
SCORCS: Shiraz Colorectal Cancer Surgery
SD: Standard deviation

## References

[1] Fitzmaurice C, Allen C, Barber RM, Barregard L, Bhutta ZA, Brenner H (2017). Global, regional, and national cancer incidence, mortality, years of life lost, years lived with disability, and disability-adjusted life-years for 32 cancer groups, 1990 to 2015: a systematic analysis for the global burden of disease study. JAMA oncology.

[2] Halimi L, Bagheri N, Hoseini B, Hashtarkhani S, Goshayeshi L, Kiani B (2020). Spatial analysis of colorectal cancer incidence in Hamadan Province, Iran: a retrospective cross-sectional study. Applied Spatial Analysis and Policy.

[3] Ahmed M (2020). Colon cancer: a clinician’s perspective in 2019. Gastroenterology research.

[4] Wong MC, Ding H, Wang J, Chan PS, Huang J (2019). Prevalence and risk factors of colorectal cancer in Asia. Intestinal research.

[5] Mansori K, Mosavi-Jarrahi A, Motlagh AG, Solaymani-Dodaran M, Salehi M, Delavari A (2018). Exploring spatial patterns of colorectal cancer in Tehran City. Iran. Asian Pacific journal of cancer prevention: APJCP.

[6] Rezaianzadeh A, Safarpour AR, Marzban M, Mohaghegh A (2015). A systematic review over the incidence of colorectal cancer in Iran. Annals of colorectal research.

[7] Riihimäki M, Hemminki A, Sundquist J, Hemminki K (2016). Patterns of metastasis in colon and rectal cancer. Scientific reports.

[8] Tamas K, Walenkamp A, De Vries E, Van Vugt M, Beets-Tan R, Van Etten B (2015). Rectal and colon cancer: Not just a different anatomic site. Cancer treatment reviews.

[9] Paschke S, Jafarov S, Staib L, Kreuser E-D, Maulbecker-Armstrong C, Roitman M (2018). Are colon and rectal cancer two different tumor entities? A proposal to abandon the term colorectal cancer. International journal of molecular sciences.

[10] Chan AT, Giovannucci EL (2010). Primary prevention of colorectal cancer. Gastroenterology.

[11] Nacion AJD, Park YY, Yang SY, Kim NK (2018). Critical and challenging issues in the surgical management of low-lying rectal cancer. Yonsei medical journal.

[12] Klaver CE, Kappen TM, Borstlap WA, Bemelman WA, Tanis PJ (2017). Laparoscopic surgery for T4 colon cancer: a systematic review and meta-analysis. Surgical endoscopy.

[13] Morneau M, Boulanger J, Charlebois P, Latulippe J-F, Lougnarath R, Thibault C (2013). Laparoscopic versus open surgery for the treatment of colorectal cancer: a literature review and recommendations from the Comité de l’évolution des pratiques en oncologie. Canadian Journal of Surgery.

[14] Rentsch M, Schiergens T, Khandoga A, Werner J (2016). Surgery for colorectal cancer-trends, developments, and future perspectives. Visceral medicine.

[15] Roeder F, Meldolesi E, Gerum S, Valentini V, Rödel C (2020). Recent advances in (chemo-) radiation therapy for rectal cancer: a comprehensive review. Radiation Oncology.

[16] Milinis K, Thornton M, Montazeri A, Rooney PS (2015). Adjuvant chemotherapy for rectal cancer: Is it needed?. World journal of clinical oncology.

[17] Fleming M, Ravula S, Tatishchev SF, Wang HL (2012). Colorectal carcinoma: Pathologic aspects. Journal of gastrointestinal oncology.

[18] Henson KE, Elliss-Brookes L, Coupland VH, Payne E, Vernon S, Rous B (2020). Data resource profile: national cancer registration dataset in England. International journal of epidemiology.

[19] Aghazadeh J, Pirnejad H, Mohebbi I, Tabrizi A, Heidari M (2019). Disease registry system in northwest of Iran: The first step forward in research progress with review of literature. Journal of Research in Clinical Medicine.

[20] Etemadi A, SAJADI A, Semnani S, NOURAEI SM, Khademi H, Bahadori M (2008). Cancer registry in Iran: a brief overview. Arch Iranian Med.

[21] Mohammadzadeh Z, Ghazisaeedi M, Nahvijou A, Kalhori SRN, Davoodi S, Zendehdel K (2017). Systematic review of hospital based cancer registries (HBCRs): necessary tool to improve quality of care in cancer patients. Asian Pacific journal of cancer prevention: APJCP.

[22] Rusch VW, Rice TW, Crowley J, Blackstone EH, Rami-Porta R, Goldstraw P. The seventh edition of the American Joint Committee on Cancer/International Union Against Cancer Staging Manuals: the new era of data-driven revisions. Mosby; 2010. 10.1016/j.jtcvs.2010.02.013 20304130

[23] Rafiemanesh H, Pakzad R, Abedi M, Kor Y, Moludi J, Towhidi F (2016). Colorectal cancer in Iran: Epidemiology and morphology trends. EXCLI journal.

[24] Feletto E, Yu XQ, Lew J-B, St John DJB, Jenkins MA, Macrae FA (2019). Trends in colon and rectal cancer incidence in Australia from 1982 to 2014: analysis of data on over 375,000 cases. Cancer Epidemiology and Prevention Biomarkers.

[25] van der Sijp MP, Bastiaannet E, Mesker WE, van der Geest LG, Breugom AJ, Steup WH (2016). Differences between colon and rectal cancer in complications, short-term survival and recurrences. International journal of colorectal disease.

[26] Deng Y. Rectal cancer in Asian vs. Western countries: why the variation in incidence? Current treatment options in oncology. 2017;18(10):1–8. 10.1007/s11864-017-0500-2 28948490

[27] Looha MA, Pourhoseingholi MA, Hosseini SV, Khodakarim S (2019). Mismeasured Covariate in the Long-Term Survival of Colorectal Cancer. Galen Medical Journal.

[28] Zare-Bandamiri M, Khanjani N, Jahani Y, Mohammadianpanah M (2016). Factors affecting survival in patients with colorectal cancer in Shiraz, Iran. Asian Pacific Journal of Cancer Prevention.

[29] Májek O, Gondos A, Jansen L, Emrich K, Holleczek B, Katalinic A (2012). Survival from colorectal cancer in Germany in the early 21st century. British journal of cancer.

[30] Yuan Y, Li M-D, Hu H-G, Dong C-X, Chen J-Q, Li X-F (2013). Prognostic and survival analysis of 837 Chinese colorectal cancer patients. World Journal of Gastroenterology: WJG.

[31] Ingeholm P, Gögenur I, Iversen LH (2016). Danish colorectal cancer group database. Clinical epidemiology.

[32] Scosyrev E, Messing J, Noyes K, Veazie P, Messing E, editors. Surveillance Epidemiology and End Results (SEER) program and population-based research in urologic oncology: an overview. Urologic Oncology: Seminars and Original Investigations; 2012: Elsevier. 10.1016/j.urolonc.2009.11.005 20363162

[33] Azizmohammad Looha M, Zarean E, Pourhoseingholi MA, Hosseini SV, Azimi T, Khodakarim S. Analyzing the long-term survival of patients with colorectal cancer: a study using parametric non-mixture cure rate models. International Journal of Cancer Management. 2018;11(9).

[34] Yin D, Morris CR, Bates JH, German RR (2011). Effect of misclassified underlying cause of death on survival estimates of colon and rectal cancer. Journal of the National Cancer Institute.

[35] Steele SR, Chang GJ, Hendren S, Weiser M, Irani J, Buie WD (2015). Practice guideline for the surveillance of patients after curative treatment of colon and rectal cancer. Diseases of the Colon & Rectum.

[36] Rawla P, Sunkara T, Barsouk A (2019). Epidemiology of colorectal cancer: Incidence, mortality, survival, and risk factors. Przeglad gastroenterologiczny.

[37] Maajani K, Khodadost M, Fattahi A, Shahrestanaki E, Pirouzi A, Khalili F (2019). Survival rate of colorectal cancer in Iran: a systematic review and meta-analysis. Asian Pacific journal of cancer prevention: APJCP.

[38] Moghadam AD, Eslami P, Razavi-Khorasani N, Moazzami B, Mousavizadeh M, Motamedi MK (2020). Colorectal cancer surgery during COVID-19 pandemic in iran; most appropriate approach. Archives of Iranian medicine.

[39] de Las Heras B, Saini KS, Boyle F, Ades F, de Azambuja E, Bozovic-Spasojevic I (2020). Cancer Treatment and Research During the COVID-19 Pandemic: Experience of the First 6 Months. Oncol Ther.

[40] Hoffmeister M, Schmitz S, Karmrodt E, Stegmaier C, Haug U, Arndt V (2010). Male sex and smoking have a larger impact on the prevalence of colorectal neoplasia than family history of colorectal cancer. Clin Gastroenterol Hepatol.

[41] Murphy G, Devesa SS, Cross AJ, Inskip PD, McGlynn KA, Cook MB (2011). Sex disparities in colorectal cancer incidence by anatomic subsite, race and age. Int J Cancer.

[42] Lin JH, Giovannucci E. Sex hormones and colorectal cancer: what have we learned so far?: Oxford University Press; 2010. 10.1093/jnci/djq444 PMC2994865 21068431

[43] Lin JH, Zhang SM, Rexrode KM, Manson JE, Chan AT, Wu K (2013). Association between sex hormones and colorectal cancer risk in men and women. Clin Gastroenterol Hepatol.

[44] Gausman V, Dornblaser D, Anand S, Hayes RB, O’Connell K, Du M (2020). Risk factors associated with early-onset colorectal cancer. Clin Gastroenterol Hepatol.

[45] Walter V, Jansen L, Hoffmeister M, Brenner H (2014). Smoking and survival of colorectal cancer patients: systematic review and meta-analysis. Ann Oncol.

[46] Gandomani HS, Aghajani M, Mohammadian-Hafshejani A, Tarazoj AA, Pouyesh V, Salehiniya H (2017). Colorectal cancer in the world: incidence, mortality and risk factors. Biomed Res Ther.

[47] Mamtani R, Cheema S, Sheikh J, Al Mulla A, Lowenfels A, Maisonneuve P (2017). Cancer risk in waterpipe smokers: a meta-analysis. Int J Public Health.

[48] Johnson CM, Wei C, Ensor JE, Smolenski DJ, Amos CI, Levin B (2013). Meta-analyses of colorectal cancer risk factors. Cancer causes & control.

[49] Luan N-N, Wu L, Gong T-T, Wang Y-L, Lin B, Wu Q-J (2015). Nonlinear reduction in risk for colorectal cancer by oral contraceptive use: a meta-analysis of epidemiological studies. Cancer Causes Control.

[50] Doubeni CA, Major JM, Laiyemo AO, Schootman M, Zauber AG, Hollenbeck AR (2012). Contribution of behavioral risk factors and obesity to socioeconomic differences in colorectal cancer incidence. J Natl Cancer Inst.

[51] Ghaem H, Amiri Z, Kianpour F, Rezaianzadeh A, Hosseini SV, Khazraei H (2016). Comparing recurrence and complications after laparoscopy and laparotomy surgery among patients suffering from colorectal cancer, Shiraz, Iran. Asian Pac J Cancer Prev.

