# Correction to: Report of 13-year survival of patients with colon and rectal cancers; lessons from Shiraz colorectal cancer surgery registry system of a level three medical center

**DOI:** 10.1186/s12893-022-01677-x

**Published:** 2022-06-24

**Authors:** Ali Reza Safarpour, Alimohammad Bananzadeh, Ahmad Izadpanah, Leila Ghahramani, Seyed Mohammad Kazem Tadayon, Faranak Bahrami, Seyed Vahid Hosseini

**Affiliations:** Colorectal Research Center, Shiraz University of Medical Sciences, Shiraz, Iran

## Correction to: BMC Surgery (2022) 22:142 https://doi.org/10.1186/s12893-022-01591-2

Following publication of the original article [1], the author name “Seyed Vahid Hosseini” was incorrectly written as “Seyed Vahid Hoseini”.

The original article has been corrected.

The online version of the original article can be found https://doi.org/10.1186/s12893-022-01591-2.
